# Inter-rater reliability of optic nerve sheath diameter measurement using real-time ultrasonography

**DOI:** 10.1186/s13089-021-00255-1

**Published:** 2022-01-10

**Authors:** Jason B. Jennings, Cynthia Oliva, Michael Joyce, Michael J. Vitto, Jordan Tozer, Lindsay A. Taylor, David P. Evans

**Affiliations:** 1 Department of Emergency Medicine, Virginia Commonwealth University, Box 980401, Richmond, VA 23298-0401 USA; 2 Department of Emergency Medicine, Medical University of South Carolina, Charleston, SC USA

**Keywords:** Optic nerve sheath diameter, Inter-rater reliability, Emergency medicine, Point-of-care ultrasound

## Abstract

**Objectives:**

Ultrasound measurement of the optic nerve sheath diameter (ONSD) is a rapid, non-invasive means to indirectly assess intracranial pressure. Previous research has demonstrated the ability of emergency physicians to measure ONSD accurately with bedside ultrasound when compared to CT scan or MRI, however the reliability of this measurement between two or more operators has been called into question (Hassen et al. in J Emerg Med 48:450–457, 2015; Shirodkar et al. in Ind J Crit Care Med 19:466–470, 2015). Given the need for accurate and precise measurement to use this as a screening exam, we sought to determine the inter-rater reliability between ONSD measurements obtained in real time by fellowship-trained emergency ultrasound physicians.

**Methods:**

Three ultrasound fellowship-trained emergency physicians measured bilateral ONSD of 10 healthy volunteers using a high-frequency linear transducer. The physicians were blinded to the other scanners’ measurements, and no instructions were given other than to obtain the ONSD. Each sonographer measured the ONSD in real time and it was recorded by a research coordinator. All measurements were recorded in millimeters. Intraclass correlation coefficients (ICCs) were calculated to estimate the inter-rater reliability.

**Results:**

A total of 60 measurements of ONSD were obtained. The average measurement was 4.3 mm (3.83–4.77). Very little variation was found between the three physicians, with a calculated ICC of 0.82 (95% confidence interval 0.63–0.92).

**Conclusions:**

ONSD measurement obtained by ultrasound fellowship-trained emergency medicine physicians is a reliable measurement with a high degree of correlation between scanners.

## Introduction

The recent spread of ultrasound technology and its adaptation at the bedside by emergency physicians has led to the exploration of a wide number of potential clinical applications [3]. Ocular ultrasonography is a relatively new application in emergency medicine. In 2002, Blaivas et al. published the first series of emergency department patients presenting with ocular symptoms who were evaluated by bedside emergency department ultrasonography [4]. The ability of ultrasound to evaluate the eye and the adjacent structures in a rapid and non-invasive manner is of tremendous value in the setting of a busy emergency department, where subspecialty consultation is not always available and physical exam may be limited by the patient’s condition. For example, a neurologic exam may be unobtainable in the unconscious, intubated and chemically paralyzed patient. The ocular exam may also be limited by severe facial and head trauma. Emergency department ultrasound provides a quick, accurate, well-tolerated and non-invasive tool for evaluating potentially vision-threatening and life-threatening conditions at the bedside [5].

Elevated intracranial pressure is a potentially devastating condition. Rapid detection and subsequent management of elevated intracranial pressure is critical as increased intracranial pressure is associated with increased morbidity, mortality and poor neurologic outcome. The gold standard for measurement of intracranial pressure is invasive and fraught with risk, requiring placement of intraventricular catheters and/or intraparenchymal probes [6].

Ultrasound measurement of the optic nerve sheath diameter (ONSD) is a rapid, non-invasive means to indirectly assess intracranial pressure [7]. The optic nerve is part of and continuous with the central nervous system via the meninges of the brain. Increases in intracranial pressure are transmitted to the retrobulbar region of the optic nerve sheath via cerebrospinal fluid. The ONSD is measured 3 mm posterior to the globe for both eyes. Though various cutoffs have been proposed, an ONSD greater than 5.8 mm is generally considered abnormal and elevated intracranial pressure should be considered [8–12]. Given the need for accurate and precise measurement to use this as a screening exam, we sought to determine the inter-rater reliability between ONSD measurements obtained in real time by fellowship-trained ultrasound physicians.

## Methods

### Study design

A prospective study with a single-measure design was used. Measurements were obtained by three ultrasound fellowship-trained emergency medicine physicians, who had each completed a one-year-long ultrasound fellowship at two different institutions. Each operator obtained ONSD measurements of both the left and right eye of each participant in either an axial or sagittal plane. The measurements took place on August 8, 2018, at the Virginia Commonwealth University Hospital. The Virginia Commonwealth University Office of Research and Innovation approved the research proposal (HM20011526).

### Participants

A convenience sample of healthy young adults was used. The sample consisted of 10 healthy volunteers recruited from the emergency medicine residency at Virginia Commonwealth University. All volunteers were informed about the study and asked to participate. Before participating, all volunteers were required to read and sign an informed consent form. The inclusion criteria for participating were the following: all volunteers were between the ages of 21–65 years and had no known medical conditions. The exclusion criteria were minors and pregnant volunteers. Ten individuals agreed to participate.

### Measurement procedures

Participants who met the inclusion criteria and agreed to participate were assessed. Three physicians performed ONSD measurements independently. All 10 participants were measured on the same day. A single measurement of the ONSD of the left and right eye of each participant was measured in the supine position using a closed-eye technique. A large amount of standard water-soluble ultrasound transmission gel was applied to the participant’s closed eyelids. The participant was asked to look straight ahead with eyes closed. The diameter was measured at 3 mm behind the globe in the transverse imaging plane using a SonoSite Xporte ultrasound machine and a high-resolution 13–6 MHz linear array ultrasound transducer (Fujifilm Bothell, WA). Each measurement was recorded in millimeters by the research coordinator.

### Statistical analyses

Intraclass correlation coefficient estimates and their 95% confidence intervals were calculated using SPSS statistical package version 25 (SPSS Inc., Chicago, IL) based on an absolute-agreement, 2-way random-effects model. An average, mean and standard deviation were also calculated.

## Results

The estimated intraclass correlation coefficient was 0.822 (0.625–0.924, 95% CI). Of the 30 measurements that were obtained, the average optic nerve sheath measurement was 4.34 mm, median was 4.30 mm and the standard deviation was 0.47. The measurements ranged from 3.5 mm to 5.2 mm.

## Discussion

In order for ocular ultrasound to serve as a sensitive screening tool in the assessment of elevated ICP, confidence and reliability of the measurement are of paramount importance. While multiple prior studies have detailed the correlation between increased ONSD and elevated ICP, no studies to our knowledge have assessed the ability of emergency physicians to obtain reliable measurements in real time. Oboerfell et al. described the ICC among a large group of emergency physicians at various stages of training measuring ONSD of static images [13]. They found an ICC among ultrasound fellowship-trained emergency physicians of 0.73, while resident emergency physicians had an ICC of 0.50. Because these measurements were based on static, previously obtained images, the ability of emergency physicians to both obtain and interpret ONSD measurements in a reliable manner was not addressed.

In our study, we assessed the ability of three ultrasound fellowship-trained emergency physicians to obtain measurements of ONSD independently and in real time on healthy volunteers. We found a high degree of measurement agreement with an ICC of 0.822. ICC is typically interpreted as optimal (>  0.8), strong (0.7–0.8), moderate (0.5–0.6), fair (0.3–0.4), or poor (0–0.2) [14, 15]. Our results suggest that ultrasound-trained emergency physicians can obtain images and measurements of the ONSD with a high degree of inter-rater reliability.

Our study is limited in its generalizability by the use of ultrasound operators who were fellowship trained in emergency ultrasound. It remains unclear how much specific training in ocular ultrasound is necessary for non-fellowship-trained physicians to accurately measure the ONSD. Additional research is necessary to assess whether non-fellowship-trained physicians can accurately image and measure the ONSD with a high degree of reliability.

## Conclusion

Ultrasonographic measurement of ONSD has the potential to serve as a non-invasive screening tool for elevated intracranial pressure. ONSD obtained by ultrasound fellowship-trained emergency medicine physicians is a reliable measurement with a high degree of correlation between scanners.

## Data Availability

Data are available for review upon request.
